# Pyroptosis in host defence against bacterial infection

**DOI:** 10.1242/dmm.049414

**Published:** 2022-07-08

**Authors:** Dominik Brokatzky, Serge Mostowy

**Affiliations:** Department of Infection Biology, London School of Hygiene and Tropical Medicine, Keppel Street, London WC1E 7HT, UK

**Keywords:** Bacterial infection, Cell-autonomous immunity, Cell death, Host-pathogen interaction, Mycobacteria, Pyroptosis, *Salmonella*, *Shigella*

## Abstract

Pyroptosis, a regulated form of pro-inflammatory cell death, is characterised by cell lysis and by the release of cytokines, damage- and pathogen-associated molecular patterns. It plays an important role during bacterial infection, where it can promote an inflammatory response and eliminate the replicative niche of intracellular pathogens. Recent work, using a variety of bacterial pathogens, has illuminated the versatility of pyroptosis, revealing unexpected and important concepts underlying host defence. In this Review, we overview the molecular mechanisms underlying pyroptosis and discuss their role in host defence, from the single cell to the whole organism. We focus on recent studies using three cellular microbiology paradigms – *Mycobacterium tuberculosis*, *Salmonella* Typhimurium and *Shigella flexneri* – that have transformed the field of pyroptosis. We compare insights discovered in tissue culture, zebrafish and mouse models, highlighting the advantages and disadvantages of using these complementary infection models to investigate pyroptosis and for modelling human infection. Moving forward, we propose that in-depth knowledge of pyroptosis obtained from complementary infection models can better inform future studies using higher vertebrates, including humans, and help develop innovative host-directed therapies to combat bacterial infection.

## Teaser

**Summary:** Pyroptosis is a form of inflammatory cell death. Here, we compare the interplay between pyroptosis and important human pathogens, i.e. *Mycobacterium tuberculosis*, *Salmonella* Typhimurium and *Shigella flexneri*, across different models of immune response to infection.

## INTRODUCTION

According to recent Nomenclature Committee on Cell Death guidelines, apoptosis and necrosis are the two main pathways of cell death (Galluzzi et al., 2018). Cells that undergo apoptosis or necrosis can be distinguished by their morphological and immunological hallmarks. In apoptotic cells, these hallmarks include cell rounding, nuclear fragmentation, formation of small vesicles – e.g. apoptotic bodies for uptake by surrounding phagocytotic cells – and their non-inflammatory potential. By contrast, hallmarks of necrotic cells include cell lysis, the absence of surrounding phagocytotic cells and their pro-inflammatory potential.

The name pyropotisis derives from the Greek words *pyro* meaning ‘fire’ or ‘fever’ and *ptosis* meaning ‘to fall’ (Cookson and Brennan, 2001; Kerr et al., 1972). Pyroptosis is a regulated cell death pathway characterised by cell lysis as well as release of pro-inflammatory cytokines, damage-associated molecular patterns (DAMPs) and pathogen-associated molecular patterns (PAMPs) (Broz et al., 2020). Pyroptosis is a regulated form of necrosis associated with inflammation and is considered part of the innate immune response in host defence (Galluzzi et al., 2018; Robinson et al., 2019). In pyroptosis, the release of DAMPs and PAMPs activates bystander cells, attracts innate immune cells and triggers an inflammatory response (Broz et al., 2020). Pyroptosis can be activated by a variety of extracellular signals, i.e. extracellular nucleotides, lipopolysaccharide (LPS), bacterial DNA and flagellin, as well as intracellular signals, i.e. oxidative stress, K^+^ efflux, mitochondrial DNA and LPS from cytosolic bacteria (Bauernfeind et al., 2010; He et al., 2016; Netea et al., 2009; Swanson et al., 2019). One hallmark of pyroptosis is formation of the inflammasome (see Box 1), whose formation can be classified as canonical or non-canonical (Box 2) (Kayagaki et al., 2015; Knodler et al., 2014; Martinon et al., 2002; Muñoz-Planillo et al., 2013; Shi et al., 2014). Pyroptosis resulting from canonical inflammasome formation depends on a priming and an activation signal. To form a priming signal, membrane receptors, such as Toll-like receptors (TLRs), are stimulated to transduce a signal that initiates expression of pyroptosis-related proteins – including NOD-like receptors (NLRs; Box 1) and pro-inflammatory cytokines, i.e.interleukin-1 beta (IL-1β) and interleukin 18 (IL-18) – through the nuclear factor kappa B (NF-κB) complex (Bauernfeind et al., 2010). The activation signal for pyroptosis is the recognition of a second intracellular signal by NLRs, such as NLRP3 (Box 1), resulting in the formation of canonical inflammasomes (Gram et al., 2021; Iyer et al., 2013; Platnich and Muruve, 2019; Zhao et al., 2011). Inflammasome formation is required for caspase-1 (CASP1) activation and, consequently, for caspase-1-dependent maturation of pro-IL-1β and gasdermin D (GSDMD) (Agostini et al., 2004; Hilbi et al., 1997; Shi et al., 2015). It is followed by the formation of GSDMD pores at the plasma membrane, which ultimately promote the release of IL-1β and pyroptosis itself (Liu et al., 2016; Xia et al., 2021). A recent study identified the 16 kDa cell-surface protein NINJ1 as mediator of plasma membrane rupture during pyroptosis (Kayagaki et al., 2021), underscoring the concept that cell death-related plasma membrane rupture is not a passive event.

In contrast to canonical inflammasome activation, the non-canonical inflammasome activation pathway results in activation of GSDMD by human caspase-4 (CASP4) and mouse caspase-11 (homologue of human caspase-4) independent of inflammasome formation. Here, intracellular LPS can be detected by inflammatory caspases that then directly cleave GSDMD, allowing it to form plasma membrane pores (Hagar et al., 2013; Kayagaki et al., 2015; Shi et al., 2014, 2015); potassium ion (K^+^) efflux through these GSDMD pores can lead to inflammasome formation, caspase-1 activation and IL-1β maturation (Rühl and Broz, 2015). The role of caspase-11 in host defence is cell-type-dependent (Kumari et al., 2021). As shown following injection of LPS into mice, activation of caspase-11 in epithelial cells does not have a significant role in inflammatory responses. By contrast, activation of caspase-11 in macrophages induces a cytokine storm and is responsible for LPS-induced shock (Kumari et al., 2021).

An in-depth understanding of the key factors and mechanisms involved in pyroptosis is starting to emerge (Broz et al., 2020; Kim et al., 2021; Swanson et al., 2019) but the precise role of pyroptosis regarding host defence and infection control remains poorly understood. The field of infection biology has contributed significantly to the discovery and characterisation of pyroptosis, highlighted by early studies of macrophage cell death during infection by *Shigella flexneri* (Zychlinsky et al., 1992). In this Review, we focus on three important cellular microbiology paradigms – *Mycobacterium tuberculosis*, *Salmonella enterica* serovar Typhimurium and *S. flexneri* – that have recently advanced our understanding of pyroptosis. All three are substantial bacterial pathogens in humans and have increasingly been studied regarding their ability to influence pyroptotic signalling pathways. *M. tuberculosis*, *S.* Typhimurium and *S. flexneri* can infect different cell types. Investigation of these different pathogens, therefore, provides the opportunity to compare molecular mechanisms of pyroptosis in both epithelial cells and macrophages. Furthermore, these pathogens have been studied in a variety of *in vitro* and *in vivo* infection models (Table 1). Drawing comparisons across these different pathogens and infection models has, therefore, the potential to transform our understanding of pyroptosis and its role in host defence. Here, we discuss molecular mechanisms of pyroptosis as discovered while investigating complementary infection models, i.e. tissue culture cells, zebrafish and mice, and highlight fundamental roles of pyroptosis in host defence, ranging from single cells to the whole organism.

**Box 1. Glossary**

**Absent in melanoma 2 (AIM2):** Host cell cytosolic innate immune receptor of double-stranded DNA recruited to form an inflammasome.

**ASC:** Apoptosis-associated speck-like protein containing a CARD (officially known as PYCARD).

**ASC specks:** Aggregates of ASC protein. ASC specks are a hallmark of inflammasome formation, assembled in host cells following activation of NLR and important for caspase-1 recruitment.

**Autophagy:** Evolutionarily conserved cytosolic degradation process important for cell-autonomous immunity.

**E3 ubiquitin ligase:** An enzyme that facilitates the transfer of ubiquitin from a ubiquitin-conjugating enzyme (E2) to the target protein.

**Granuloma:** Cluster of immune cells within inflamed tissue, a hallmark of mycobacterial infection in the lung.

**Guanylate-binding proteins (GBPs):** Host cell family of interferon-inducible GTPases that bind to bacterial surfaces to trigger the activation of an inflammasome. This way, GBPs restrict the replication of intracellular pathogens in both immune and non-immune cells.

**Inflammasome:** Host cell cytosolic protein complex responsible for activation of caspase-1, inflammation and pyroptosis.

**NACHT, LRR and PYD domains-containing protein 3 (NLRP3):** Broad-spectrum cytosolic innate immune receptor of damage- and pathogen-associated molecular patterns known for its ability to form an inflammasome in host cells.

**NLR family apoptosis inhibitory protein (NAIP):** Anti-apoptotic human protein that inhibits the cysteine proteases CASP3, CASP7 and CASP9, and functions as the sensor component of the NAIP/NLRC4 inflammasome.

**NLR family CARD domain-containing protein 4 (NLRC4):** Member of the NLR protein family known for its ability to form an inflammasome in host cells by interacting with NAIP.

**Nucleotide-binding oligomerisation domain-like receptors (NLRs; also known as nucleotide oligomerisation domain (NOD)-like receptors:** Family of cytosolic innate immune receptors responsible for host cell immune responses, such as NF-κB activation or inflammasome formation, following the recognition of diverse damage- and pathogen-associated molecular patterns.

**O-antigen:** Highly variable component of the lipopolysaccharide on the surface of Gram-negative bacteria. The O-antigen is a key bacterial molecule involved in host–pathogen interactions.

**Outer membrane vesicles (OMVs):** Produced by Gram-negative bacteria to provide DNA or nutrients to other bacteria or transmit virulence factors to the host cell.

***Salmonella* containing vacuole (SCV):** Modified phagosome and replicative niche for *Salmonella* post invasion of host cells.

**Septins:** Unconventional GTP-binding cytoskeletal proteins that assemble in host cells to form filaments, bundles, rings and cage-like structures.

**Transposon-directed insertion-site sequencing (TraDIS):** High-throughput technique combining genome-wide transposon mutagenesis with high-throughput sequencing; providing insight into the function and/or essentiality of genetic components in a bacterial genome.

**Type III secretion system (T3SS):** Secretion machinery found in Gram-negative bacteria, important for host cell invasion and virulence.

**Type VII secretion system (T7SS):** Secretion machinery found in Gram-positive bacteria, important for bacterial physiology, interbacterial competition and virulence.

**Wiskott–Aldrich syndrome (WAS):** Genetic disorder caused by mutation of WASP actin nucleation promoting factor (WAS, also known as WASp) that results in hyperinflammation and immune deficiency.

**Box 2. Molecular mechanisms of pyroptosis**

The canonical inflammasome is a cytosolic protein complex consisting of a NOD-like receptor (NLR), such as NLRP3, a caspase-recruitment domain (CARD)-containing protein, such as ASC (Box 2), and caspase-1 (Agostini et al., 2004; Martinon et al., 2002; Vajjhala et al., 2012). In the absence of CARD-containing proteins, NLRP3 requires the adaptor protein ASC for inflammasome assembly and caspase-1 activation (Broz et al., 2010; Suzuki et al., 2007; Vajjhala et al., 2012). Canonical inflammasome assembly is followed by caspase-1-dependent maturation of IL-1β and GSDMD (Agostini et al., 2004; Hilbi et al., 1997; Shi et al., 2015). Mature GSDMD integrates into the plasma membrane and forms pores that promote the release of IL-1β and, ultimately, pyroptosis (Liu et al., 2016; Xia et al., 2021).

Canonical formation of the NAIP/NLRC4 inflammasome is triggerd by intracellular flagellin (Zhao et al., 2011). In this case, NAIP recognizes the bacterial protein flagellin as its ligand and, together with NLRC4, forms an inflammasome that serves as an activation platform for caspase-1. Active caspase-1 catalyses the maturation of IL-1β and GSDMD, leading to pyroptosis (Kofoed and Vance, 2011; Zhang et al., 2015).

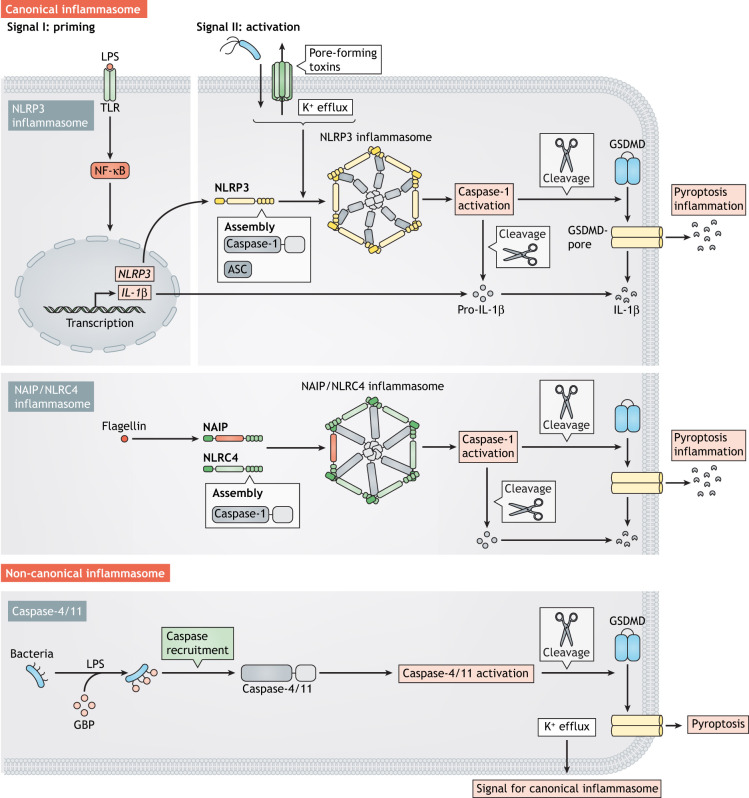

In contrast to canonical inflammasome formation, non-canonical inflammasome formation results in activation of GSDMD by caspase-4 in humans and by caspase-11 in mice. Prior to activation, synthesis of caspase-11 is induced by an upstream stimulus by, e.g. type-I interferons (Rathinam et al., 2012). Whereas caspase-1 is activated by canonical inflammasome formation, caspase-4/11 is activated by recognition of bacterial LPS, resulting in the maturation of GSDMD (Kayagaki et al., 2015; Shi et al., 2014, 2015). Mature GSDMD then forms pores that facilitate K^+^ efflux, leading to canonical inflammasome formation, caspase-1 activation, IL-1β maturation and pyroptosis (Kayagaki et al., 2015; Rühl and Broz, 2015; Shi et al., 2015; Xia et al., 2021).

**Table 1.**
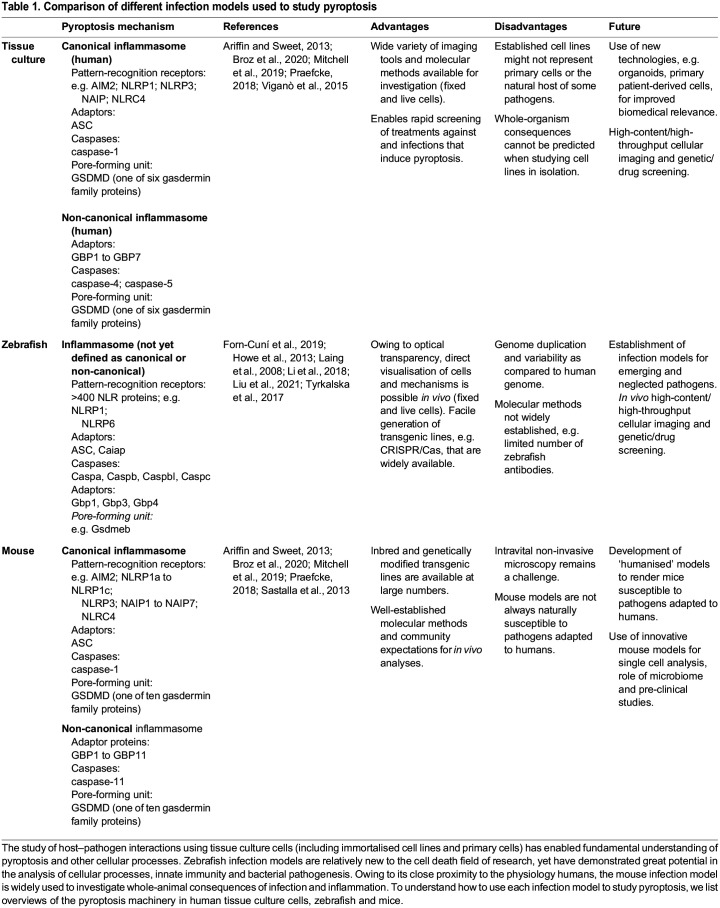
Comparison of different infection models used to study pyroptosis

## The interplay between mycobacteria and pyroptosis

In 2020, 1.5 million people died of tuberculosis and 200,000 people were infected with drug-resistant *M. tuberculosis*, the causative agent of tuberculosis in humans (WHO, 2021). Alarmingly, tuberculosis death rates are increasing because of the SARS-CoV-2 pandemic and restricted hospital access (WHO, 2021). *M. tuberculosis* is a facultative intracellular pathogen known to infect a variety of cell types, including epithelial cells and macrophages, and is widely recognised for forming granulomas (Box 1) during its infection cycle (Castro-Garza et al., 2002; Huang et al., 2020; Pai et al., 2016; Ryndak and Laal, 2019; Thacker et al., 2020).

### Activation of pyroptosis by mycobacteria

The interplay between *M. tuberculosis* and pyroptosis has been the subject of intense investigation. A breakthrough result was the observation that *M. tuberculosis* infection of IL-1β-deficient mice showed increased bacterial burden and mortality compared to infected wild-type mice (Mayer-Barber et al., 2010). These data suggested that IL-1β secretion and signalling and, therefore, inflammasome formation, has an essential role in the control of mycobacterial infection. Consistent with this hypothesis, induction of the NLRP3 inflammasome has often been reported during *M. tuberculosis* infection of mice (Carlsson et al., 2010; Dorhoi et al., 2012; McElvania Tekippe et al., 2010). Next, we focus on more-recent studies investigating the activation of pyroptosis by *M. tuberculosis*.

**Fig. 1.**
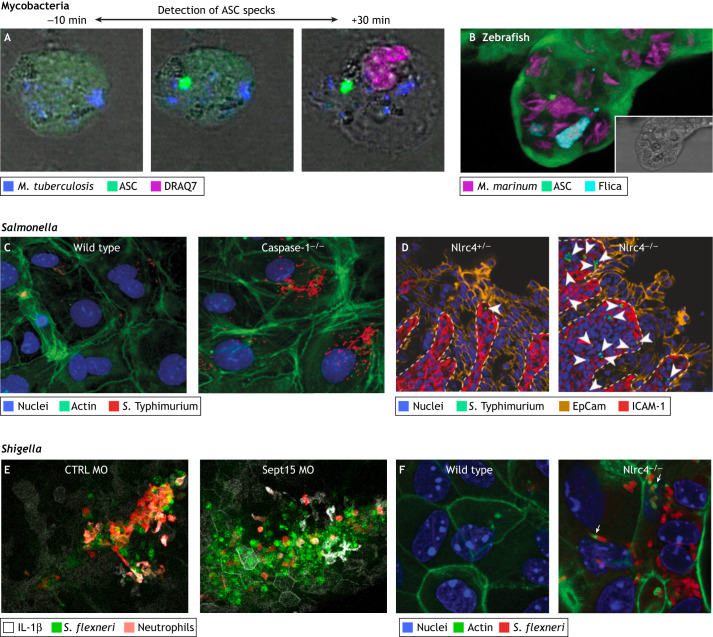
**Different infection models to investigate pyroptosis induced by mycobacteria, *Salmonella* or *Shigella*.** (A) Representative time-lapse microscopy images of a THP-1 macrophage expressing GFP-tagged ASC, infected with *M. tuberculosis* (blue). Shown is a pyroptotic cell death event of an infected cell. Left image: ASC specks, a hallmark of inflammasome formation, are shown in green. Right image: uptake of the DNA dye DRAQ7 (red), was used to detect cell death. The images show the induction of pyroptosis in human macrophages within 40 minutes after infection with *M. tuberculosis* and illustrate how dying cells become permeabilised, as shown by the uptake of DRAQ7. ASC speck formation can be detected when *M. tuberculosis* is intracellular. Figure panels adapted from Beckwith et al. (2020). (B) Confocal and bright-field (inset) microscopy image of a granuloma in a zebrafish larva 3 days post infection with *M. marinum* (red). ASC specks are shown in green. Active caspases were labelled using a Flica assay based on a fluorescent inhibitor probe (FAM-YVAD-FMK) with Caspa shown in blue (Flica). Figure panel adapted from Varela et al. (2019 preprint). (C) Confocal microscopy images of wild-type and caspase-1-deficient mice cecal enteroid monolayers infected with mCherry-expressing *S*. Typhimurium (red). Increased intracellular bacterial burden can be seen in the caspase-1-deficient (Caspase-1^−/−^) cells. In the absence of caspase-1, the epithelial layer fails to control bacterial proliferation, highlighting caspase-1 as a crucial factor in cell-autonomous immunity. These results demonstrate the importance of caspase-1 activation for infection control in *Salmonella*-infected epithelial cells. Figure panels adapted from Crowley et al. (2020). (D) Confocal microscopy images of *S.* Typhimurium-infected murine intestine showing the significantly increased bacterial burden in the lamina propria of NLRC4-deficient (NLRC 4^−/−^) mice at 48 hours post infection compared with that of heterozygous (NLRC 4^+/−^) control mice. EpCAM staining (orange) highlights the presence of epithelial cells, iCAM-1 staining (red) indicates endothelium, arrowheads indicate infection with *S.* Typhimurium (green). In the absence of the NLRC4 inflammasome, mice fail to control infection with *Salmonella* and this image highlights the role of NLRC4 in controlling infection *in vivo*. The location of the lamina propria is indicated by dashed lines. EpCAM, epithelial cell adhesion molecule (EPCAM); iCAM-1, intercellular adhesion molecule 1. Figure panels adapted from Fattinger et al. (2021). (E) Time-lapse confocal microscopy images of Tg(*il-1b*:GFP-F) x Tg(*lyz*:dsRed) zebrafish larvae injected with either control or *sept15*-targeting morpholino oligonucleotides (CTRL MO or Sept15 MO, respectively), followed by infection of the hindbrain ventricle for 19 hours with E2-Crimson-expressing *S. flexneri* M90T. Notice that lack of Sept15 significantly increased the bacterial burden (see staining for *S. flexneri*, dark green) and inflammation (see staining for neutrophils, red), and indicated by expression of IL-1β (white). The *Shigella*-zebrafish infection model enables investigation of the cell biology of infection *in vivo*, in this case showing increased cytokine expression and immune cell recruitment during *S. flexneri* infection. Zebrafish larvae are optically accessible, allowing non-invasive imaging of cellular events *in vivo* at high resolution. Figure panels adapted from Mazon-Moya et al. (2017). (F) Confocal microscopy images of wild-type or 129.NLRC4-deficient (NLRC 4^−/−^) cecum-derived mouse intestinal epithelial cells grown in a monolayer and infected with *S. flexneri* (red). Here, Mitchell et al. discovered that, in the absence of the NAIP/NLRC4 inflammasome, mice develop a shigellosis-like phenotype. The images show that *S. flexneri* can replicate and form actin tails (arrows) in NLRC 4^−/−^ murine epithelial cells. Figure panels adapted from Mitchell et al. (2020).

Induction of pyroptosis by *M. tuberculosis* has recently been further investigated using human and mouse macrophages (Mohareer et al., 2018; Patrick and Watson, 2021). *M. tuberculosis* infection of the human acute monocytic leukaemia-derived monocyte cell line THP-1 cells has shown that inflammasome activation and pyroptosis depend on the mycobacterial type VII secretion system (T7SS; Box 1**)** called ESAT-6 secretion system 1 (also known as and hereafter referred to as secretion system ESX-1) (Fig. 1A) (Beckwith et al., 2020). Here, when mycobacteria infect macrophages, ESX-1-dependent induction of phagosomal damage leads to K^+^ efflux and formation of the NLRP3 inflammasome. Consistent with this, when macrophages are infected with an ESX-1-mutant strain of *M. tuberculosis*, formation of ASC specks (Box 1**)** and secretion of IL-1β is significantly reduced compared to infection with wild-type bacteria (Beckwith et al., 2020; Wassermann et al., 2015). Together, these studies show that *M. tuberculosis* can induce pyroptosis in macrophages and suggest that pyroptosis is important for successful infection in humans. Although not yet clear *in vivo*, it is tempting to speculate that formation of the NLRP3 inflammasome in response to the ESX-1 secretion system promotes bacterial dissemination. In support of this hypothesis, time-lapse imaging of infected THP-1 cells has shown that ∼25% of *M. tuberculosis* are released from pyroptotic cells, promoting bacterial dissemination (Beckwith et al., 2020).

A recent study examined the role of macrophage pyroptosis during infection with *M. tuberculosis* by using immortalised bone marrow-derived macrophages (iBMDMs) from C57/BL6 mice, showing that genetic or pharmacological inhibition of the inflammasome significantly reduces bacterial survival (Subbarao et al., 2020). These data showed that blocking the NLRP3 inflammasome, either by deletion of inflammasome components or by drug treatment with the inhibitor MCC950, significantly reduces IL-1β release and bacterial burden.

**Fig. 2.**
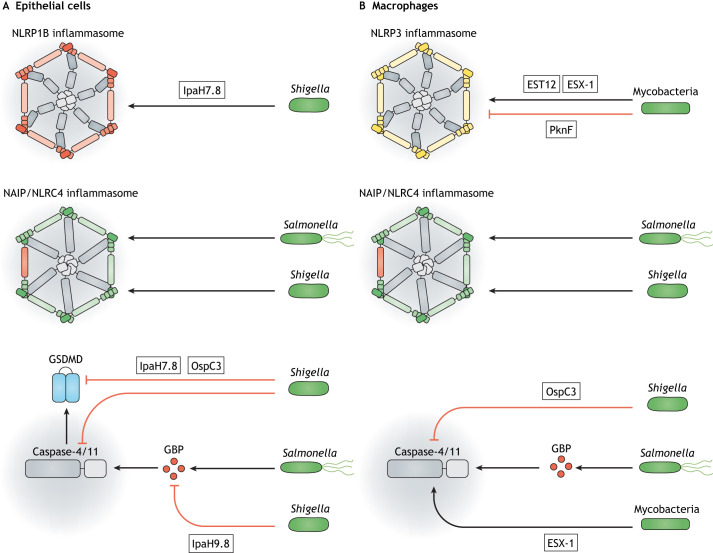
**Bacterial interactions with the inflammasome.** (A) Top: Bacterial interactions with the inflammasome in epithelial cells. IpaH7.8 is an E3 ubiquitin ligase expressed by *Shigella* (Sandstrom et al., 2019), which can activate murine NLRP1B by ubiquitylating its N-terminus and targeting it to proteasomal degradation. Middle: Following the detection of *Salmonella* and *Shigella* by NAIP, NLRC4 is recruited to form the inflammasome (Broz, 2015; Fattinger et al., 2021; Gram et al., 2021; Mitchell et al., 2020). Bottom: IpaH7.8 also targets human gasdermin D (GSDMD) for proteasome degradation and, this way, can block pyroptosis of human but not mouse cells (Luchetti et al., 2021). The *Shigella* effector arginine ADP-riboxanase (OspC3) blocks the activation of caspase-4/11 (Kobayashi et al., 2013; Li et al., 2021a) and uses the effector IpaH9.8 to block GBP-mediated bacterial recognition (Li et al., 2017; Wandel et al., 2017). However, *Salmonella* is recognised by GBPs and this initiates the recruitment and activation of caspase-4, followed by GSDMD maturation and pyroptosis (Santos et al., 2020). (B) Top: Bacterial interactions with the inflammasome in macrophages. *M. tuberculosis* induce ESX-1-dependent phagosomal damage, leading to K^+^ efflux and formation of the NLRP3 inflammasome (Beckwith et al., 2020). This process can also be induced by EST-12 (Qu et al., 2020). However, *M. tuberculosis* can also inhibit the NLRP3 inflammasome via the bacterial phosphokinase PknF (Rastogi et al., 2021). Middle: The NAIP/NLRC4 inflammasome also recognises bacterial OMVs from *Salmonella* (Yang et al., 2020). In mouse macrophages, *Shigella*-induced cell death depends on NLRC4 (Mitchell et al., 2020). Bottom: The *Shigella* effector OspC3 blocks the activation of caspase-4/-11 (Kobayashi et al., 2013; Li et al., 2021a). During *Salmonella* infection, GBP-mediated recognition of bacteria is important for induction of pyroptosis (Fisch et al., 2019). In infected murine macrophages, induction of pyroptosis by *M. marinum* depends on caspase-11 (Varela et al., 2019 preprint). EST12, cell pyroptosis-inducing protein in *M. tuberculosis* (officially known as Rv1579c); ESX-1, ESAT-6 secretion system 1; NLRP1B, NACHT, LRR and PYD domain-containing protein 1b allele 1 (*Mus musculus*).

A preprint by Varela and colleagues showed that extracellular DNA from *Mycobacterium marinum*, a natural fish pathogen closely related to *M. tuberculosis* (Hashish et al., 2018), stimulates pyroptosis in zebrafish larvae and in murine macrophages (Fig. 1B) (Varela et al., 2019 preprint). In the murine macrophage cell line RAW264.7, pathogen-induced pyroptosis depends on caspase-11 and GSDMD. Infection of RAW264.7 cells with a *M. marinum* RD1 mutant, which lacks the ESX-1 system, did not induce pyroptosis, recapitulating the observations in *M. tuberculosis* infection of human monocytes (Fig. 2) (Varela et al., 2019 preprint; Wassermann et al., 2015). Four caspase-1-like genes have been annotated in zebrafish, i.e. *caspa*, *caspb*, *caspbI* and *caspc* (Forn-Cuní et al., 2019; Varela et al., 2019 preprint). Infections of Caspa-deficient zebrafish with the RD1 mutant did not affect bacterial burden compared to infection of wild-type zebrafish, whereas burden of wild-type bacteria was reduced in Caspa-deficient zebrafish (Varela et al., 2019 preprint). Together, these results suggest that bacterial escape from the phagosome is essential for pyroptosis activation. In zebrafish, the same preprint by Varela and colleagues showed that Caspa is activated during *M. marinum* infection and that activation of Gasdermin Eb (Gsdmeb) – a zebrafish protein with a GSDM domain and caspase-1 cleavage site – through Caspa promotes bacterial release by killing the host cell. Expression of mouse caspase-11, but not mouse caspase-1, in Caspa-deficient zebrafish was able to replace Caspa function, indicating that Caspa-dependent Gsdmeb activation is comparable to caspase-11 activation in mice. By contrast, activation of an ASC-dependent pathway via Caspb controls bacterial infection. Here, the Caspb-dependent IL-1β response appears to be important for *M. marinum* control, as shown by the increase of bacterial burden in infected Caspb-, IL-1β- or ASC-deficient larvae as compared to control larvae. These data suggest that activation of different pyroptotic pathways can result in different infection outcomes for the host. Whereas activation of the non-canonical inflammasome pathway, which depends on Caspa and Gsdmeb, is pro-bacterial, activation of the canonical inflammasome pathway, which depends on Caspb and ASC, is anti-bacterial. Further investigation of pro- versus anti-bacterial outcomes when studying canonical versus non-canonical inflammasome activation in zebrafish and other infection models is, therefore, of great interest.

By testing 40 different secreted *M. tuberculosis* proteins for their ability to induce death of the host cell, Qu and colleagues identified Rv1579c (also known and hereafter referred to as EST12) as a pyroptosis-inducing protein (Fig. 2) (Qu et al., 2020). In macrophages, EST12 binds to receptor for activated C-kinase 1 (RACK1), leading to activation of NLRP3 and formation of inflammasomes. Recent work proposed that RACK1, together with the mitotic Ser/Thr protein kinase NEK7, triggers a conformational change in NLRP3 required to promote inflammasome formation (Duan et al., 2020). Understanding the precise role of RACK1 in NLRP3 inflammasome formation may be important to control *M. tuberculosis* infection in humans.

### Inhibition of pyroptosis by mycobacteria

*M. tuberculosis* can also inhibit pyroptosis. For example, coinfection of bone marrow-derived dendritic cells (BMDCs) with *M. tuberculosis* and the avirulent *Mycobacterium smegmatis* has shown that inhibition of the AIM2 (Box 1) inflammasome by *M. tuberculosis* depends on ESX-1 (Shah et al., 2013; Sharma et al., 2019). Infection of BMDCs with *M. smegmatis* can induce pyroptosis; yet, co-infection experiments revealed that wild-type *M. tuberculosis* can block the secretion of IL-1β (Shah et al., 2013). When these coinfection experiments were performed by using an *M. tuberculosis* mutant deficient for EsxA, a factor secreted by the ESX-1 secretion system, the secretion of IL-1β induced by *M. smegmatis* was not blocked (Shah et al., 2013). Together, these results suggest that, during infection of mouse dendritic cells, factors secreted by *M. tuberculosis* can inhibit the formation of the AIM2 inflammasome and pyroptosis.

Studies in mouse BMDMs have revealed that *M. tuberculosis* can block pyroptosis by inhibiting the NLRP3 inflammasome (Rastogi et al., 2021). The bacterial factors involved in the inhibition of pyroptosis are the subject of intense investigation and, in the case of *M. tuberculosis*, inhibition of the NLRP3 inflammasome is dependent on the bacterial phosphokinase PknF (Fig. 2) (Rastogi et al., 2021). BMDMs infected with PknF-deficient mutant mycobacteria showed significantly increased IL-1β secretion and cell death compared to macrophages infected with wild-type bacteria. Strikingly, cells infected with *M. tuberculosis* were resistant to inflammasome activation induced by LPS and the antibiotic nigericin, whereas PknF-deficient bacteria failed to block inflammasome activation, IL-1β secretion and cell death. These data suggest that PknF plays an important role in blocking inflammasome activation during infection. Considering that PknF has been reported to control *M. tuberculosis* growth (Deol et al., 2005), this unexpected role for PknF in pyroptosis induction may suggest that bacterial growth can be a crucial trigger for inflammasome formation.

Overall, these studies show that mycobacteria can inhibit pyroptosis. A better understanding of these processes *in vivo* might suggest alternatives to the traditional treatment using antibiotics during mycobacterial infection of humans. Recent evidence suggests that cell death in *M. tuberculosis*-infected mouse macrophages is independent of caspase-1 and caspase-11 (Zhang et al., 2021). However, a type I IFN-dependent regulation of cell death plays an important role in host defence against mycobacterial infections (Zhang et al., 2021). In this case, RAW264.7 macrophages deficient for the IFN receptor IFNAR2 show reduced cell death upon *M. tuberculosis* infection compared that of wild-type RAW264.7. The interplay of type I IFN signal transduction and cell death shows that different inflammatory pathways can influence each other, and that studying the interactions between different inflammatory pathways might illuminate new strategies for infection control.

## Pyroptosis in host defence against *Salmonella* infection

*Salmonella enterica*, a Gram-negative bacterium, is the causative agent of gastroenteritis (Bäumler et al., 1998). Research of *S*. *enterica* serovar Typhimurium has contributed significantly to the field of cellular microbiology and to host-pathogen interactions (reviewed in López-Jiménez and Mostowy, 2021), and this pathogen is often used for studies of host cell death induced by bacterial infection (Bierschenk et al., 2017; Boise and Collins, 2001; Wemyss and Pearson, 2019). Moreover, the dynamic ability of *S.* Typhimurium to infect both epithelial cells and macrophages enables the comparison of cell death mechanisms in different cell types (Bäumler et al., 1998; Galán, 2021; Kihlstrom, 1977; Weinstein et al., 1984).

### Activation of pyroptosis by Salmonella

Recent work using C57BL/6 mice and cecal enteroids has shown that both the canonical and non-canonical inflammasome pathways (Box 2) are important in the control of *S.* Typhimurium infection in intestinal epithelial cells (Crowley et al., 2020). C57BL/6 mice deficient for caspase-1 and caspase-11 and, therefore, unable to trigger pyroptosis, cannot control bacteria, which highlights the importance of epithelial cell pyroptosis as a host defence mechanism (Fig. 1C). Recent work has shown that the barrier of the epithelial NLR family apoptosis inhibitory protein (NAIP) and the NLR family CARD domain-containing protein 4 (NLRC4) (Box 1) protects mice from a tumour necrosis factor (TNF)-driven inflammatory response during *S.* Typhimurium infection (Fig. 1D) (Fattinger et al., 2021). Here, the NAIP/NLRC4 inflammasome (Box 2) appears to be anti-bacterial and a host defence mechanism that helps in bacterial control. A possible mechanistic explanation is that NAIP/NLRC4 inflammasome-mediated pyroptosis eliminates the replicative niche for *Salmonella.*

Some NLRs, including NLRC4, can directly recruit caspase-1 via their caspase-recruitment domain (CARD), and activate it without ASC. In this case, NLRC4 is recruited to form the inflammasome following NAIP-mediated detection of the *Salmonella* flagellin (Fig. 2) (Broz, 2015; Gram et al., 2021). However, it was previously shown that, although NLRC4 recruitment of caspase-1 without ASC does lead to cell death, it also leads to inefficient cytokine processing and secretion (Broz et al., 2010). Considering that NLRC4 can independently recruit caspase-1, the precise role of ASC in the formation of the NAIP/NLRC4 inflammasome and pyroptosis induction remains of great interest.

In addition to triggering pyroptosis in response to intracellular bacteria, work in BMDMs has shown that the NLRC4 inflammasome can also recognise bacterial outer membrane vesicles (OMVs; Box 1; Fig. 2) (Yang et al., 2020). Considering that flagellated OMVs, i.e. those that carry bacterial flagellin to the host cell, released by *S.* Typhimurium can activate the NLRC4 inflammasome, it has been proposed that NAIP5, a sensor of bacterial flagellin, interacts with NLRC4 to promote inflammasome assembly and pyroptosis (Fig. 2) (Yang et al., 2020; Zhao et al., 2011). However, the expression of FilC, a flagellar subunit, is downregulated during *S.* Typhimurium infection as measured in spleens of mice (Cummings et al., 2005), highlighting that the role of flagellated OMVs *in vivo* is still unclear. Despite this, the ability of host cells to react to OMVs demonstrates how extracellular bacteria can be recognised by an intracellular recognition system, suggesting that manipulating this recognition can be useful as a therapy against *Salmonella* infection.

Studies have shown that guanylate-binding proteins (GBPs; Box 1) are host factors involved in bacterial recognition and pyroptosis activation (Fisch et al., 2019; Santos et al., 2020; Wandel et al., 2020). GBP1 plays a key role in caspase-4 activation during *S.* Typhimurium infection of THP-1 cells. Here, GBP1 binds to the *Salmonella*-containing vacuole (SCV; Box 1), enables caspase-4 recruitment to the vacuole and promotes caspase-4 activation (Fig. 2) (Fisch et al., 2019). Work in HeLa cells has shown that GBPs can bind to *S.* Typhimurium LPS and activate pyroptosis once bacteria have escaped the SCV (Santos et al., 2020). In HeLa cells stimulated by the type II interferon IFN-γ, GBPs can assemble into signalling platforms at the surface of Gram-negative bacteria for activation of caspase-4. These GBPs can recognize cytosolic bacteria, such as *Salmonella*, to promote recruitment and activation of caspase-4, maturation of GSDMD and pyroptosis (Santos et al., 2020).

Our understanding of pyroptosis during bacterial infection and its systemic consequences becomes more complex when considering that short-chain fatty acids (SCFAs) produced by gut microbiota can influence macrophage pyroptosis (Tsugawa et al., 2020). Experiments in the human monocyte cell line U937 showed that SCFAs can bind to the PYRIN domain of NLRs and to ASC, and that this binding enhances inflammasome formation in human monocytes. In *Salmonella* infection of BMDMs, SCFAs facilitate bacterial clearance by promoting inflammasome formation and pyroptosis. Wild-type or ASC-deficient C57BL/6 mice treated with antibiotics – therefore, rendering them microbiota free – showed no difference in susceptibility to *Salmonella* infection. However, the survival of ASC-deficient mice not treated with antibiotics was significantly reduced compared to untreated wild-type mice, suggesting that SCFAs produced by the microbiota promote ASC-dependent pyroptosis and host defence (Tsugawa et al., 2020). It is, thus, of great interest to study the interplay between microbiota and the induction of pyroptosis during infection. Considering that first-line antibiotic treatment disrupts the normal gut microbiome, resulting in the loss of SCFA-dependent induction of pyroptosis that may be helpful in clearing infection, improved understanding of the normal gut microbiome and its role during pyroptosis holds therapeutic promise for treatment of *Salmonella*. SCFAs have been described to suppress the growth of T3SS-expressing bacteria (Hockenberry et al., 2021) and, therefore, it is still unclear whether the effect of SCFAs on pyroptosis is direct, i.e. acting on the host cell, or indirect, i.e. a result of bacterial elimination.

The highly host-adapted *S*. *enterica* serovar Typhi causes typhoid fever in humans (Johnson et al., 2018a). Despite its many similarities to *S.* Typhimurium, infection of THP-1 cells with *S.* Typhi triggers increased caspase-1 cleavage, IL-1β secretion and cell death – in contrast to infection with *S.* Typhimurium (Lin et al., 2020). Activation of pyroptosis by *S.* Typhi is associated with expression of the *Salmonella* pathogenicity island 1 (SPI-1), which carries genes essential for growth and virulence.

The expression of SPI-1 genes (e.g. *sipC*, *sipD*, *sopB*) and the stability of SPI-1-encoded proteins from *S.* Typhi are different from those of *S.* Typhimurium. For example, in *S.* Typhi as compared to *S.* Typhimurium, the half-life of HilD, a main transcriptional regulator of SPI-1, is longer and the expression of some SPI-1-encoded genes is increased following stimulation with bile (Johnson et al., 2018b).

Expression of the bacterial virulence gene *sipD* that encodes the tip protein part of the type III secretion system (T3SS; Box 1**)** (Chatterjee et al., 2011), the *Salmonella* invasion gene *invA* (Galan et al., 1992), and *hilA* that encodes the transcription factor regulating expression of SPI-1-encoded genes (Lostroh et al., 2000), was increased in *S.* Typhi compared to *S.* Typhimurium, and might account for increased pyroptosis. Consistent with this hypothesis, THP-1 cells infected with SipD-, InvA- or HilA-deficient bacteria show significantly reduced IL-1β secretion and cell death compared to cells infected with wild-type bacteria (Lin et al., 2020).

In summary, both *S.* Typhimurium and *S.* Typhi can induce pyroptosis upon entering host cells. However, *S.* Typhi can induce pyroptosis more potently than *S.* Typhimurium, highlighting an increased potential of this serovar to activate cell death during human infection. *S.* Typhi virulence genes have been identified by using transposon-directed insertion site sequencing (TraDIS; Box 1**)** and infection of a humanised mouse model, for which mice were engrafted with human immune cells (Karlinsey et al., 2019). However, further studies are required to fully understand the pathogenic differences between *S.* Typhimurium and *S.* Typhi, the whole-organism consequences of increased pyroptosis induction by *S.* Typhi, and whether these mechanisms can uncover therapeutic windows to treat gastroenteritis and typhoid fever.

### Inhibition of pyroptosis by *Salmonella*

*S*. Typhimurium has also been shown to inhibit pyroptosis *in vivo*. The *Salmonella* plasmid virulence C (*spvC*) gene can inhibit pyroptosis during infection of C57BL/6 mice (Zuo et al., 2020). SpvC is closely related to the *Shigella* virulence factor OspF, a phosphorthreonine lyase involved in dephosphorylation of mitogen-activated protein kinase (MAPK) pathway components (Li, 2007; Mazurkiewicz et al., 2008). As compared to the ceca of mice infected with wild-type *S*. Typhimurium, ceca of mice infected with SpvC-deficient bacteria showed increased levels of NLRP3 and NLRC4. In the same study, GSDMD cleavage was increased during infection of J774A.1 cells and of murine ceca by SpvC-deficient bacteria as compared to infection with wild-type *S*. Typhimurium (Zuo et al., 2020). In addition to demonstrating the role of SpvC in pyroptosis inhibition, the same study showed the importance of SpvC for bacterial dissemination in mice and damage to secondary tissues, such as spleen and liver, during infection. These findings indicate that downregulation of pyroptosis by the virulence factor SpvC is essential for *S.* Typhimurium pathogenesis.

## *Shigella* – a paradigm for inflammatory cell death

*Shigella* is the causative agent of bacterial dysentery and responsible for ∼164,000 deaths per year (Kotloff et al., 2018). For over three decades, various *Shigella* infection models have promoted our understanding of both infection and cell biology (reviewed in Duggan and Mostowy, 2018; Schnupf and Sansonetti, 2019). *Shigella* significantly contributed to our understanding of infection-induced cell death, highlighted in 1992 by a seminal study pre-dating the term ‘pyroptosis’, which showed that *Shigella* induces DNA fragmentation and macrophage cell death (Zychlinsky et al., 1992).

### Activation of pyroptosis by *Shigella*

Considering that a hyperinflammatory response to *Shigella* and many other pathogens can be detrimental to the host, the inflammatory response must be tightly regulated (Tan et al., 2021). Septins (Box 1) are enigmatic cytoskeletal proteins that can interact with other cytoskeletal components, including actin and microtubules, and cellular membranes. Septins have been implicated in a wide variety of cellular processes, including cell shape and movement (Mostowy and Cossart, 2012; Spiliotis and Gladfelter, 2012), and are widely recognised for their roles in cell division and host defence (Hartwell, 1971; Mostowy and Cossart, 2012; Mostowy et al., 2010; Robertin and Mostowy, 2020; Van Ngo and Mostowy, 2019). By primarily using HeLa cells, several groups have shown that septins can entrap actin-polymerising *Shigella* in cage-like structures for targeting to autophagy (Box 1) (Krokowski et al., 2018; Lobato-Márquez et al., 2021; Mostowy et al., 2010; Sirianni et al., 2016). Myeloid cells derived from Wiskott–Aldrich syndrome (WAS; Box 1) patients exhibit increased inflammasome assembly, IL-1β response and cell death upon pyroptotic stimulation with LPS and ATP/nigericin (Lee et al., 2017). The WASP actin nucleation promoting factor (WAS, hereafter referred as WASp) regulates the actin cytoskeleton and influences the NF-κB pathway by facilitating NF-κB complex activation and translocation to the nucleus (Huang et al., 2005; Ngoenkam et al., 2021). During *Shigella* infection, WASp deficiency significantly reduced septin caging and bacterial clearance in BMDCs (Lee et al., 2017). During infection of THP-1 cells in which WASp was depleted via shRNA-mediated knockdown, IL-1β secretion and bacterial replication was increased compared to control cells with unperturbed levels of WASp. Consistent with this, WASp-deficient BMDCs infected with enteropathogenic *E. coli*, a relative of *Shigella*, showed decreased colocalisation of SEPT2, and autophagy markers p62 and LC3 with intracellular bacteria (Lee et al., 2017). Together, these results indicate that WASp deficiency can reduce septin caging and antibacterial autophagy, therefore, increasing IL-1β-mediated inflammation and bacterial survival within the host. The underlying mechanism by which WASp regulates the autophagy machinery is still poorly understood. However, it is tempting to speculate that the loss of WASp increases pyroptosis-dependent inflammation due to its lack of regulation by autophagy, actin and septins.

Using zebrafish to investigate the role of septins in inflammation during *Shigella* infection *in vivo* (Fig. 1E), Mazon-Moya and colleagues discovered that septin dysfunction may be an underlying factor in hyperinflammation, highlighting a novel role for septins in host defence against bacterial infections. They observed increased levels of caspase-1 activity and cell death in the absence of Sept15 (the zebrafish homologue of human SEPT7), and the survival of infected zebrafish was significantly reduced compared to controls (Mazon-Moya et al., 2017). These results suggest that, in the absence of the septin cytoskeleton, cells are less resistant to infection, resulting in hyperinflammation and reduced host survival (Mazon-Moya et al., 2017; Mostowy and Shenoy, 2015).

Further work showed that *S. flexneri*-induced death of THP-1 cells is increased compared with that induced by *Shigella sonnei* (Watson et al., 2019), a pathogen responsible for shigellosis, an infection primarily found in developed countries (Holt et al., 2012; Torraca et al., 2020). The reduction of pyroptosis was attributed to the O-antigen (Box 1) in *S. sonnei* and its reduced ability to invade THP-1 cells*.* Although induction of cell death by *S. sonnei* is reduced compared with that by *S. flexneri*, in both cases induction of cell death strictly depends on their T3SS. Surprisingly, infection of zebrafish revealed that *S. sonnei* is significantly more virulent *in vivo* than *S. flexneri*, and that the *S. sonnei* O-antigen is primarily responsible for neutrophil cell death during infection (Torraca et al., 2019). The precise mode of neutrophil cell death during *S. sonnei* infection is unknown but by experimentally infecting zebrafish with *Edwardsiella piscicida*, a fish pathogen of the *Enterobacteriaceae* family (Leung et al., 2019), showed that neutrophils, indeed, undergo pyroptosis (Chen et al., 2021).

Bacterial factors from several pathogens can activate pyroptosis (Chui et al., 2019; Fischer et al., 2020; Lin et al., 2020; Sandstrom et al., 2019). One important example is IpaH7.8 (Fig. 2), the *Shigella* E3 ubiquitin ligase (Box 1) (Sandstrom et al., 2019). IpaH7.8 can activate mouse NACHT, LRR and PYD domain-containing protein 1b allele 1 (NLRP1B), leading to inflammasome formation and caspase-1 activation. Inactive NLRP1B is autoinhibited through its N-terminal domain. IpaH7.8 ubiquitylates this N-terminus and marks it for proteasomal degradation, which – in turn – releases the NLRP1B C-terminal fragment that can assemble into the inflammasome following recruitment of caspase-1 (Chui et al., 2019; Sandstrom et al., 2019). Ubiquitylation of NLRP1B by IpaH7.8 is a landmark example of direct activation of an intracellular receptor by a bacterial enzyme. This activation leads to inflammasome formation and is a host defence mechanism crucial for the recognition of secreted bacterial factors in the cytosol.

It has been challenging to find an animal model for the pathogenesis of shigellosis and there is no natural mouse model for *Shigella* infection (McGuire and Floyd, 1958; Schnupf and Sansonetti, 2019). Strikingly, infecting mice with *Shigella* triggers an inflammasome response that protects them from shigellosis. However, recent data showed that C57BL/6J, C57BL/6N and 129S1/SvImJ mice that lack the NAIP/NLRC4 inflammasome can develop shigellosis-like phenotypes (Mitchell et al., 2020). Mitchell et al. also showed in murine intestinal epithelial cells that recognition of bacteria by the NAIP/NLRC4 inflammasome, followed by inflammasome and pyroptosis induction are sufficient to protect the mice from *S. flexneri* infection (Fig. 2) (Mitchell et al., 2020). The authors suggested that *S. flexneri* can trigger a mechanism to inhibit or evade NAIP/NLRC4 inflammasome recognition in humans but not in mice, in which the inflammasome within intestinal epithelial cells provides a barrier that protects from disease (Fig. 1F). Cell death induced by *S. flexneri* was independent of NLRC4 in human THP-1 cells, whereas mouse BMDMs lacking NLRC4 did not undergo pyroptosis (Fig. 2). By contrast, the ability of mouse epithelial cells to control *S. flexneri* (Mitchell et al., 2020) as well as *S.* Typhimurium infection (Fattinger et al., 2021) via the NAIP/NLRC4 inflammasome, highlights the importance of the epithelial inflammasome in host defence. Considering that NAIP/NLRC4-deficient mice are susceptible to shigellosis, the cell-autonomous immune response driven by the NAIP/NLRC4 inflammasome is probably a main factor underlying the resistance of wild-type mice to *S. flexneri* infection.

As shown by using human epithelial (HeLa and 293T kidney) and endothelial (Ea.hy92) cell lines, bacterial IpaH7.8 can target human GSDMD for proteasome degradation, whereas this function is blocked in mouse epithelial cells (Fig. 2) (Luchetti et al., 2021). This means that the ability of mouse epithelial cells to detect infection via the NAIP/NLRC4 inflammasome pathway is crucial to prevent bacterial virulence. In NLRC4-deficient C57BL6/N mice, GSDMD has been shown to promote the resistance to *S. flexneri* infection by blocking bacterial replication (Luchetti et al., 2021). Compared to GSDMD-deficient mice, deletion of NLRC4 alone showed an increase in tissue damage and weight loss during infection but no significant increase in the bacterial burden. However, deletion of both NLRC4 and GSDMD significantly increased bacterial replication in intestinal tissue and susceptibility to *S. flexneri*, both of which progressed to bloody diarrhoea. Together, these elegant *in vivo* studies revealed that cell-autonomous immunity by NLRC4/GSDMD-mediated pyroptosis in the epithelial cells of the intestinal tract protects wild-type mice against *S. flexneri* infection (Mitchell et al., 2020; Luchetti et al., 2021). The translational implications of this protection are not yet clear, given the natural susceptibility of humans to shigellosis. However, it is interesting to consider the evolutionary origins of pyroptosis against *Shigella*, and that inflammasome-deficient mouse models now offer the possibility to test therapeutic treatments against shigellosis.

Overall, the induction of pyroptosis by *Shigella* can be viewed as both anti- and pro-bacterial (Ashida et al., 2021). The ability of epithelial cells to induce pyroptosis can eliminate the intracellular niche of *Shigella* and, thus, act as an anti-bacterial mechanism, whereas pyroptosis in macrophages can promote bacterial dissemination in the pro-bacterial host and can, therefore, be seen as pro-bacterial. Both bacterial and host factors regulate pyroptosis during *Shigella* infection, yet the precise role of different host cell types in host defence, including epithelial cells and macrophages, requires further investigation.

### Inhibition of pyroptosis by *Shigella*

As highlighted by the above-mentioned bacterial E3 ubiquitin ligase IpaH7.8 that targets human GSDMD for proteasome degradation (Luchetti et al., 2021), *Shigella* can inhibit pyroptosis. In contrast to the canonical NAIP/NLRC4 inflammasome, the non-canonical inflammasome pathway (Box 2) does not protect mice against shigellosis (Li et al., 2021a,b). In this case, *S. flexneri* can block caspase-11-mediated detection of LPS via its bacterial effector OspC3 (Fig. 2). The potential of OspC3 to inhibit caspase-4 in human epithelial cells was described almost 10 years ago (Kobayashi et al., 2013). OspC3 belongs to the OspC family of effectors secreted by the T3SS (Buchrieser et al., 2000; Le Gall et al., 2005). Remarkably, OspC3 can block activation of caspase-11 by an only recently described post-translational modification called adenosine diphosphate (ADP)-riboxanation, in which OspC3 catalyses ADP addition to as well as deamination of arginine in a nicotinamide adenine dinucleotide (NAD^+^)-dependent manner (Li et al., 2021a,b). These findings highlight the use of *S. flexneri* to discover unexpected ways in which bacterial pathogens can evade pyroptosis, and to reveal fundamental processes in cell biology. By using *Shigella* as a paradigm, a new post-translational modification – i.e. ADP-riboxanation – that is likely to also have important roles in uninfected cells, was identified and confirmed the importance of tightly regulated inflammasome formation in pyroptosis.

As described above for *Salmonella* infection, GBP-mediated recognition of cytosolic bacteria leads to recruitment and activation of caspase-4, GSDMD maturation and pyroptotic cell death (Finethy et al., 2017; Santos et al., 2020; Shi et al., 2014; Wandel et al., 2020). Unlike *Salmonella*, *Shigella* can evade this cell-autonomous immune pathway by secreting a bacterial effector called OspC3 (Kobayashi et al., 2013; Kutsch et al., 2020; Li et al., 2017). OspC3 can inhibit caspase-4 by binding to its p19 subunit. To counteract GBP binding, *Shigella* express the E3 ubiquitin ligase IpaH9.8 that ubiquitylates GBPs and targets them for proteasomal degradation (Fig. 2) (Li et al., 2017; Wandel et al., 2017, 2020). Consequently, IpaH9.8 can be viewed as a bacterial factor that prevents GBP-mediated host defence. In this way, the avoidance of GBP recognition by *Shigella* interferes with pyroptosis by removing the platform required for caspase-4 activation.

## Conclusions

In this Review, we propose that deeper understanding of the interactions between bacterial pathogens and pyroptosis in a variety of infection models is important to comprehensively understand infections and inflammation in humans. Although we focus on three pathogens, *M. tuberculosis*, *S.* Typhimurium and *S. flexneri*, future studies need to investigate neglected and emerging bacterial species. This should include both laboratory-adapted strains as well as clinical isolates and will increase the repertoire of bacterial pathogens available to understand the precise roles of pyroptosis in host defence and their translational potential.

Considering the evolution of inflammasome components (Devant et al., 2021; Digby et al., 2021; Johnson et al., 2021), it will be exciting to investigate bacterial-induced pyroptosis across different species – e.g. zebrafish versus mouse versus human – to discover evolutionarily conserved and unique pathways of host defence (see Box 3). For the first time, newly developed technologies, such as microfluidic chips and organoids allow researchers to more fully capture the biomedical relevance of these pathways (López-Jiménez and Mostowy, 2021). Considering that, historically, investigations of host cell death relied on human cancer cell lines, such as HeLa and THP-1 – neither of which are the most physiologically relevant model cell lines – it is important to now apply innovative, more biologically relevant, modelling technologies to the field of infection biology and cell death. Future work should compare inflammasome formation and pyroptosis induction by using different cell types, pathogen species and innovative technologies, to understand the full breadth of mechanisms and outcomes underlying the interactions between bacterial pathogens and pyroptosis. Such mechanistic insights will one day illuminate if and where inhibition of this pro-inflammatory cell death pathway can be used to counteract infection in humans.

**Box 3. Outstanding questions.**

• How can bacterial pathogens be controlled by manipulating pyroptosis? Should host-directed therapies aiming to combat bacterial infection and antimicrobial resistance have a pro- or anti-pyroptotic effect?

• In epithelial cells and macrophages, what are the different triggers and consequences of pyroptosis? In the intestine, how can these differences be used to control *Salmonella* or *Shigella* infection?

• Does the role of pyroptosis in host defence depend on the infected cell type? Will targeting pyroptosis as a host-directed therapy only work by targeting specific cell types?

• What is the crosstalk between pyroptosis and other cell-death pathways during bacterial infections? How would this crosstalk interfere with therapeutic strategies aiming to only target pyroptosis?

• Studying components and pathways of pyroptosis from diverse host organisms will yield evolutionary insights and define general roles for pyroptosis in antibacterial immunity. What can research using animal models tell us about the role pyroptosis has in human infection?
